# Correction: Favipiravir and/or nitazoxanide: a randomized, double-blind, placebo-controlled trial of early therapy in COVID-19 in health workers, their household members, and patients treated at IMSS (FANTAZE)

**DOI:** 10.1186/s13063-023-07364-3

**Published:** 2023-05-22

**Authors:** Tania A. Smith, Carlos Hoyo-Vadillo, Akosua Agyeman Adom, Liliana Favari-Perozzi, Silke Gastine, Hakim-Moulay Dehbi, Beatriz Villegas-Lara, Eduardo Mateos, Yessica Sara Pérez González, Maria D. Navarro-Gualito, Alejandra S. Cruz-Carbajal, Miguel A. Cortes-Vazquez, Carolina Bekker-Méndez, Charmina Aguirre-Alvarado, Gisela Aguirre-Gil, Lucero Delgado-Pastelin, Andrew Owen, David Lowe, Joseph Standing, Jorge Escobedo

**Affiliations:** 1 CINVESTAV-IPN, Mexico, Mexico; 2 ICH-UCL, London, UK; 3 Clinical Trials Unit, UCL, London, UK; 4 IMSS, Mexico, Mexico; 5 Hakken Enterprise, Cuernavaca, Mexico; 6 University of Liverpool, Liverpool, UK; 7 Division of Infection and Immunity, UCL, London, UK

**Correction: Trials 23, 583 (2022)**

**https://doi.org/10.1186/s13063-022-06533-0**

The original publication of this article [1] erroneously contained "2X2 design" in the title whilst the study itself did not include a 2 × 2 design. The original article has been updated.
